# Effects of Isomaltulose and Gamma-Irradiated Taro Flour on Selected Physicochemical Properties and Consumer Acceptance of Pudding

**DOI:** 10.3390/foods14193350

**Published:** 2025-09-26

**Authors:** Suteera Vatthanakul, Napassorn Salamun, Tatcha Cheersomsuk, Pumnat Chuenchomrat, Philipda Suthipibul, Surasak Sajjabut, Witoon Prinyawiwatkul

**Affiliations:** 1 Department of Food Science and Technology, Faculty of Science and Technology, Thammasat University, Pathumthani 12121, Thailand; napassorn.sala@dome.tu.ac.th (N.S.); tatcha.che@dome.tu.ac.th (T.C.); cpumnat@tu.ac.th (P.C.); s.philipda@gmail.com (P.S.); 2 Thammasat University Center of Excellence in Food Science and Innovation, Pathumthani 12121, Thailand; 3 Thailand Institute of Nuclear Technology (Public Organization), 9/9 Moo 7, Saimoon, Nakhon Nayok 26120, Thailand; surasak@tint.or.th; 4 School of Nutrition and Food Sciences, Louisiana State University Agricultural Center, Baton Rouge, LA 70803-4200, USA; wprinya@lsu.edu

**Keywords:** pudding, isomaltulose, taro flour, gamma irradiation, syneresis, volatile profile, consumer acceptance

## Abstract

The quality of pudding using different types of sugar (sucrose at 5% by weight or isomaltulose 5% or 10% by weight) in a formulation was studied. Adding isomaltulose resulted in less water being separated (syneresis) from the pudding structure after 15 days of storage and increased texture firmness. The pudding product containing 10% isomaltulose received the highest scores for consumer acceptance for texture, taste, and overall liking (7.00–7.60; moderately to very much liked). The effects of gamma irradiation at different doses (0, 2, 4, and 6 kGy) on taro flour were studied. All doses of irradiation did not significantly (*p* > 0.05) affect the proximate chemical composition of taro flour. The irradiation dose used to treat taro flour significantly affected (*p* ≤ 0.05) the syneresis of the puddings, with increasing doses decreasing the observed syneresis after 15 days of storage while increasing texture firmness. The effects of gamma irradiation on taro flour at 6 kGy resulted in a more pleasant odor, including sweet (toluene), jasmine/sweet (2-cyclopenten-1-one), almond (benzaldehyde), and nutty (2-methyl-3-methylthio-pyrazine) in the pudding sample. Furthermore, such a sample was the most liked (7.30) compared to other pudding samples. This study demonstrated that isomaltulose and irradiated taro flour could be used to produce pudding samples with desirable quality and sensory liking.

## 1. Introduction

Taro is an agricultural root crop in Thailand. It can be cultivated all year round, which makes it a popular choice for consumption. It is used in both savory and sweet dishes. The flesh is pale purple with a distinct aroma [1]. Taro corms are rich in carbohydrates, fiber, vitamins, and beneficial minerals. For instance, 100 g of taro provides 112 kilocalories, 26.46 g of carbohydrates, 1.5 g of protein, and 0.2 g of fat [2]. Taro has a glycemic index (GI) of 51, making it a food with a low glycemic index. This may make it suitable for diabetic patients and individuals who want to control their weight. Beyond its GI value, taro contains resistant starch and high dietary fiber content, which slow glucose absorption and contribute to improved blood sugar control. In addition, bioactive compounds such as flavonoids, alkaloids, saponins, and tannins present in taro have been associated with hypoglycemic effects. Taro is also rich in essential micronutrients, including vitamins C and B complex, beta-carotene, iron, and folic acid, which promote overall health. These nutritional characteristics support the potential of taro as a dietary option for individuals managing diabetes or aiming to maintain healthy blood glucose levels [3]. Additionally, taro is free from gluten protein, which is beneficial for certain groups, such as those with gluten allergies or celiac disease. In addition, taro is also affordable and readily available compared to wheat flour, which often needs to be imported in many countries. However, despite its nutritional benefits and being a good energy source, unprocessed taro contains oxalates and phytates, which bind to other minerals such as calcium, iron, and phosphorus, making the body unable to absorb them. According to Alcantara et al. [4], when taro was processed into flour, the oxalate and phytate content decreased compared to raw taro. Oxalic acid content decreased when taro was processed or heated. Taro starch has been used as a gelling agent, thickener, and stabilizer in various food products such as bread, noodles, cookies, and pudding [4,5].

Currently, there is a growing trend towards health consciousness and managing one’s health. Moreover, puddings are a popular type of dessert that is easy to consume and widely enjoyed. The main ingredients in pudding production are milk and sugar. Consequently, consumers who aim to maintain their health often avoid consuming puddings because high sugar intake can lead to elevated blood sugar levels, increasing the risk of heart disease and diabetes. Therefore, there have been attempts to alter pudding recipes to cater to health-conscious consumers, including the use of alternative ingredients to replace refined sugar. Isomaltulose, having a GI of 38, is classified as a low-GI ingredient (GI less than 55), whose GI is twice as low as that of regular sugar [6]. Isomaltulose is metabolized and absorbed slowly in the body after consumption, making it suitable for those with diabetes or health-conscious individuals. Therefore, pudding formulations made with isomaltulose could address the needs of consumers who prioritize health, offering a dessert option that minimizes the rapid increase in blood sugar levels. According to Kobayashi et al. [7], the blood sugar levels in healthy subjects who consumed isomaltulose were lower than those who consumed sucrose. They also tested individuals with type 2 diabetes who consumed either isomaltulose or sucrose and examined the postprandial changes in blood sugar levels. They reported that blood sugar levels increased rapidly after consuming sucrose but increased gradually after consuming isomaltulose. Therefore, using isomaltulose instead of sucrose to develop pudding products for health purposes presents another interesting option.

Another common issue with pudding products during storage is syneresis, which refers to the undesirable separation of liquid from the gel structure on the pudding’s surface. Gamma irradiation, a food preservation method, helps adjust the chemical and physical properties of food products. Currently, gamma irradiation is utilized in various research fields, such as studying the swelling and viscosity of legume plants. Research data have indicated that radiation technology can improve the structure of starch by enhancing its water-holding capacity. According to Theprugsa et al. [8], gamma irradiation technology may help delay syneresis in pudding products. Therefore, it has been proposed to combine taro flour with radiation technology to reduce syneresis issues and enhance the product’s value.

Therefore, this research aimed to develop pudding products using isomaltulose and taro flour that was gamma-irradiated at different doses in order to improve some physicochemical properties, the volatile profile, and the consumer acceptance of pudding products.

## 2. Materials and Methods

### 2.1. Pudding Preparation

The ingredients used to make the pudding included taro flour (Gosenka Co., Ltd., Bangkok, Thailand), water, lactose-free milk (CP-Meiji Co., Ltd., Saraburi, Thailand), sucrose (Mitr Phol Sugar Co., Ltd., Suphan Buri, Thailand), isomaltulose (Rajburi Sugar Co., Ltd., Kanchanaburi, Thailand), and agar (Seng Huad Co., Ltd., Bangkok, Thailand). All ingredients, as shown in Table 1, were manually mixed, then brought to a temperature of 80 °C for 15 min while constantly being stirred manually using a whisk. Afterward, the mixture was poured into 2 × 2 × 2 m^3^ cups and cooled down in a refrigerator at 4 °C for 8 h.

### 2.2. Gamma Irradiation and Proximate Chemical Composition of Taro Flour

The gamma irradiation treatment on taro flour was performed at the Thailand Institute of Nuclear Technology (TINT; Ongkharak, Nakhon Nayok, Thailand). Taro flour was irradiated at 0, 2, 4, and 6 kGy by using a ^60^Co source. Moisture, fat, protein, and ash contents (%) were determined according to the AOAC method [9]. Total carbohydrate content (%) was calculated by subtracting the total amount of moisture, fat, protein, and ash contents from 100.

### 2.3. Determination of Physicochemical Properties of Puddings

Analyses of color, pH, and total soluble solids (TSS).

The color profile of the pudding samples was determined using a colorimeter (CX2687, Hunter Lab, Reston, VA, USA). The values of lightness (L*), redness (a*), and yellowness (b*) were measured.

The pH of pudding sample solution after boiling was measured at room temperature (25 °C) using a pH meter (PB-20, Sartorius, Göttingen, Germany).

The TSS of pudding sample solution after boiling were measured at room temperature (25 °C) using a refractometer (MASTER-2PM, ATAGO, Tokyo, Japan).

Texture Profile Analysis.

The pudding samples were removed from the refrigerator and immediately tested at refrigerated temperature (4 °C) for texture using Texture Analyser (TA-XT2i, Stable Micro Systems, Godalming, UK) using a cylindrical probe (50 mm diameter) to compress the 2 × 2 × 2 cm^3^ pudding sample to 50% of its height at a speed of 5.00 mm/s. The test speed was 0.25 mm/s, the post-test speed was 5.00 mm/s, and the trigger force was 6 g for all pudding samples [10]. Each sample underwent two compression cycles. The textural parameters (hardness, Springiness, cohesiveness, and gumminess) were quantified from the TPA curves.

Syneresis.

Syneresis measurement was conducted by following the method of Eadmusik et al. [11] with modifications. Pudding samples were contained in sealed plastic cups and stored in the refrigerator at 4 °C for 1 and 15 days. The pudding sample was drained by pouring the water that separated from the pudding structure and weighed. The syneresis was calculated by

(1) $$\%\mathrm{Syneresis}=\frac{\left(initial\;weight\;of\;pudding\;sample-drained\;weight\;of\;pudding\;sample\right)}{initial\;weight\;of\;pudding\;sample\;}\times 100$$

### 2.4. Microbial Analysis of Pudding Samples

The total plate count in pudding products containing taro flour irradiated at 0 and 6 kGy was measured by Asia Medical and Agricultural laboratory and Research Center (AMARC) using the FDA-BAM online method [12]. Additionally, yeasts and molds in these pudding products were measured by Asia Medical and Agricultural laboratory and Research Center (AMARC) using the AOAC method [13].

### 2.5. Consumer Acceptance Test

The sensory evaluation protocol was approved by the Human Research Ethics Committee of Thammasat University, approval no. 68SC018. Consumer acceptance tests of pudding samples were performed by 60 untrained panelists aged between 18 and 25 years. Two sets of pudding samples were as follows: Set I. Samples containing sugar, 5% isomaltulose, and 10% isomaltulose. Set II. Samples containing taro flour irradiated at 0, 2, 4, and 6 KGy. The pudding serving quantity was 25 g per cup. Each cup was coded with a three-digit random code for serving. Attribute parameters (appearance, color, flavor, texture, taste, and overall liking) were evaluated on a 9-point hedonic scale (1 = dislike extremely; 5 = neither like nor dislike; and 9 = like extremely).

### 2.6. Volatile Profile Analysis by GC-MS

The thin-film solid-phase micro-extraction (TF-SPME) of PDMS/DVB (20 mm × 4.65 mm, 90 µm phase thickness) was used to absorbed volatiles from taro flours irradiated at 0, 2, 4, and 6 kGy and pudding products made with taro flours irradiated at 0 and 6 kGy. Five grams of each sample was placed into a 20 mL glass vial. Then a screw cap with TF-SPME samples and its holder was tightened over headspace. To absorb volatiles from the sample, the vial was incubated at 40 °C for 20 min without shaking. TF-SPME samples were placed into a TDU glass tube and robotically inserted onto the thermal desorption unit (TDU; GERSTEL, Mülheim an der Ruhr, Germany) of GC-MS (Agilent, 8890 GC System, Shanghai, P.R. Chaina and 5977B MSD, Agilent Technologies, Inc. CA, USA). Volatiles were desorbed at 150 °C for 1 min and separated by an HP-5 MS column (30 m, 0.25 mm ID, and 0.25 μm) with 1 mL/min He carrier gas. The temperature programming conditions were as follows: the initial temperature was 35 °C, which was kept for 4 min, and subsequently, the temperature was increased at 16 °C/min up to 160 °C, and then it was further increased at 25 °C/min to 250 °C and held for 4 min. MS conditions were 230 °C, 70 eV, 35–300 a.m.u. scanning, and NIST20 library.

### 2.7. Statistical Analysis

This study was designed with a Completely Randomized Design (CRD) to analyze TSS, pH, and texture and syneresis, while an experiment with a Randomized Complete Block Design (RCBD) was used to analyze consumer acceptance. This experiment was based on the mean values of two independent replicates. An analysis of data variance (ANOVA) and Duncan’s New Multiple Range Test were performed to determine statistical differences at *p* < 0.05.

## 3. Results and Discussion

### 3.1. The Effects of Different Types of Sugar on the Physicochemical Properties and Consumer Acceptance of Puddings

In this study, two types of sugars were used: sucrose (S) at 5% and isomaltulose (I) at 5% and 10%. Herein, 10% isomaltulose was selected due to its sweetness, which is approximately 50% that of sucrose. The total soluble solids (TSS) and pH of pudding products containing sucrose (S), 5% isomaltulose (5I), and 10% isomaltulose (10I) were 16.50, 15.50, and 21.50 ^o^Brix and 6.54, 6.13, and 6.51, respectively (Table 2). It was observed that TSS significantly increased as the amount of sugar or isomaltulose increased from 5% to 10%. Specifically, the pudding sample with 10% isomaltulose had approximately 1.3 times more dissolved solids than the other two studied samples. In contrast, the pH values (Table 2) and color values (Table 3) of the three pudding samples were not significantly different, which was also reported by Hadjikinova [14] for instant creams made with sugar or isomaltulose.

The syneresis of pudding samples on the first day of storage was not significantly different (Table 4). However, after 15 days of storage, the samples were significantly different; the pudding containing isomaltulose exhibited significantly less syneresis compared to the pudding containing sucrose. These findings are consistent with the results reported by Hadjikinova [14]. The findings from this study revealed significant insights into the role of isomaltulose in enhancing pudding stability by minimizing water separation. This effect can be attributed to the hydroxyl groups present in the sugar’s molecular structure, which effectively bind water through hydrogen bonds [15], thereby maintaining product integrity over time. Moreover, the α-(1→6) linkage between glucose and fructose in isomaltulose, compared to the α-(1→2) linkage in sucrose, provides higher acid stability and slower hydrolysis, helping to maintain a stable concentration of soluble solids. This maintains a consistent osmotic balance, minimizes water migration within the gel, and reduces syneresis. Additionally, the stable crystal structure of isomaltulose with bound water further supports water retention in the pudding matrix [16].

The textural hardness and cohesiveness of the pudding products were significantly different due to the types of sugar used (Table 5). The pudding containing 10% isomaltulose had the highest hardness value (915.93) but the lowest cohesiveness value (0.12). The enhanced syneresis (water-binding capability) likely contributed to the observed increase in the hardness value. The gel structure becomes stronger as it binds effectively more with water. However, this improvement was accompanied by a decrease in cohesiveness because the sugar could have disrupted the gel structure, making it less resistant to strain [17].

Different types of sugar did not have significant effects on liking scores for the color and flavor of pudding products (Table 6). However, there were significant differences in liking scores for appearance, texture, taste, and overall liking with the pudding containing 10% isomaltulose (10I) receiving the highest score (7.0–7.7; moderately to very much liked). The findings were aligned with previous research demonstrating similar benefits in other confections [18]. Such findings suggested that isomaltulose could be a potential ingredient in improving the consumer acceptance of various food items. Therefore, based on these favorable results, a pudding product containing 10% isomaltulose was chosen for studying the effects of gamma irradiation on the taro flour used in pudding products.

### 3.2. The Effects of Gamma Irradiation on the Proximate Composition of Taro Flour

The moisture, fat, protein, and ash contents of taro flour treated with different doses of gamma irradiation (0, 2, 4, and 6 kGy) were not significantly different (Table 7). Bashir et al. [19] and Bashir and Aggarwal [20] reported similar results for whole wheat flour and chickpea flour, respectively.

### 3.3. The Effects of Gamma Irradiation on the Volatile Profile of Taro Flour and Puddings

**Figure 1:**
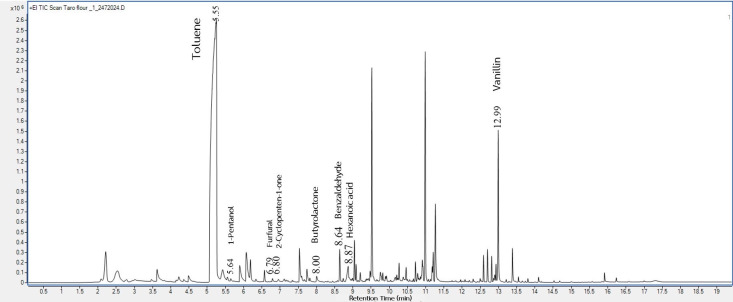
The representative volatile profile of non-irradiated taro flour.

**Figure 2:**
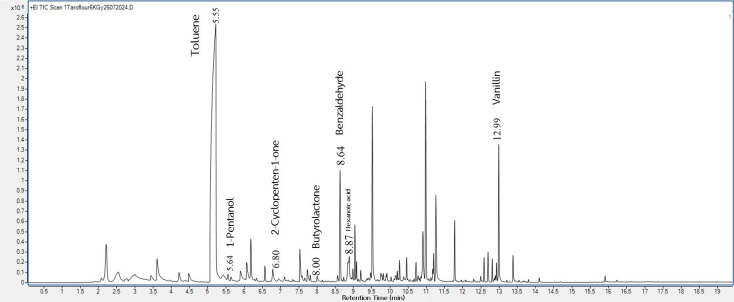
The representative volatile profile of taro flour irradiated at a dose of 6 kGy.

**Figure 3:**
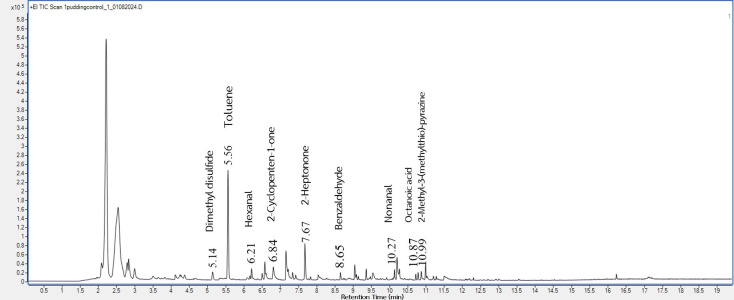
The representative volatile profile in a pudding sample containing non-irradiated taro flour.

**Figure 4:**
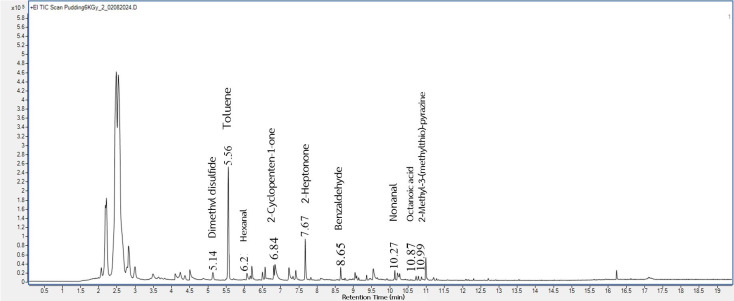
The representative volatile profile in a pudding sample containing taro flour gamma-irradiated at a dose of 6 KGy.

This study used GC-MS to identify the volatile compounds from the taro flours before and after gamma irradiation at various dosages. These irradiated taro flours were used in the pudding product formulations, and the pudding volatiles were also analyzed. There were 95 (shown in Figure 1), 99, 89, and 93 (shown in Figure 2) volatiles deriving from the headspace of 0, 2, 4, and 6 kGy irradiated taro flours, respectively. In addition, for pudding samples, a total of 44 volatile compounds were detected in the control (Figure 3), and 38 volatile compounds were in the pudding containing gamma-irradiated flour at 6 kGy (Figure 4). Some key odor compounds, their peak areas, and an odor description of gamma-irradiated taro flour and puddings containing irradiated taro flour are presented in Table 8 and Table 9.

It was observed that several volatiles with pleasant and sweet odor descriptions were detected in both non-irradiated and irradiated taro flours; these included toluene, 1-pentanol, furfural, 2-cyclopenten-1-one, butyrolactone, benzaldehyde, and vanillin (Table 8). Moreover, the contents of toluene, 1-pentanol, 2-cyclopenten-1-one, butyrolactone, and benzaldehyde increased when taro flours were irradiated at a higher dosage of gamma rays (Table 8). Hexanoic acid eliciting an unpleasant sweat and cheese odor, however, was also detected in all samples but at a lesser concentration at the 6 kGy irradiation dose (Table 8).

The representative GC-MS chromatograms of pudding products containing taro flour both non-irradiated (Figure 3) and irradiated at 6 kGy (Figure 4) show some similar pleasant volatiles (Table 9) observed in taro flours (Figure 1 and Figure 2 and Table 8). The contents of the pleasant volatiles (toluene, 2-cyclopenten-1-one, benzaldehyde, and 2-methyl-3-methylthio-pyrazine) in the pudding sample increased after the gamma irradiation of taro flour at a dose of 6 kGy (Table 9). The slightly increased fruity odor from 2-heptonone was also detected in the puddings containing irradiated taro flour. On the other hand, off-odor volatiles of dimethyl disulfide, hexanal, nonanal, and octanoic acid were also detected but decreased using irradiated taro flour. Gamma irradiation applied to other food products affected their flavors [25,26]. Flavor changes in food after irradiation were caused by various components, such as water, fat, protein, and other compounds. In the presence of irradiation, the water fraction could be broken down into hydroxyl radicals (oxidizing radicals), aqueous electrons, and hydrogen atoms (reducing compounds) [25]. These species initiated various food reactions, which affected organic molecules and altered the flavor of the irradiated taro products. The results of these volatile profiles corresponded to those of the consumer acceptance of the puddings, which will be discussed later.

**Table 9:** Volatile compounds of pudding products containing taro flours treated with different doses of gamma irradiation.

Compound	RT	Peak Area		Odor Description	References
		0 kGy	6 kGy		
Dimethyl disulfide	5.14	7.8 × 10^4^	6.3 × 10^4^	Onion	[23]
Toluene	5.56	3.5 × 10^5^	4.3 × 10^5^	Sweet	[21]
Hexanal	6.21	4.6 × 10^4^	3.1 × 10^4^	Grass, Tallow, Fat	[23]
2-Cyclopenten-1-one	6.84	ND	1.2 × 10^5^	Jasmine, Sweet	[24]
2-Heptonone	7.67	1.0 × 10^5^	1.3 × 10^5^	Fruity	[23]
Benzaldehyde	8.65	2.2 × 10^4^	3.9 × 10^4^	Almond, Nutty	[23]
Nonanal	10.27	2.6 × 10^4^	1.5 × 10^4^	Fat, Green	[23]
Octanoic acid	10.87	3.1 × 10^4^	1.4 × 10^4^	Sweat, Cheese	[23]
2-Methyl-3-(methylthio)-pyrazine	10.99	3.8 × 10^4^	6.3 × 10^4^	Nutty	[27]

Note: ND means not detected.

### 3.4. The Effects of Gamma-Irradiated Taro Flour on the Physicochemical Properties, Microbial Safety, and Consumer Acceptance of Puddings

The total soluble solids (TSS) and pH values of pudding products made with taro flour treated with different doses of gamma irradiation were not significantly different (Table 10).

The L* values (color lightness; 71.52–71.88) were not significantly different among pudding samples. However, the a* (redness) values of puddings slightly decreased with the irradiation doses at 2–4 kGy and then increased at 6kGy (Table 11); similar results were reported for strawberries [28]. Regarding the b* (yellowness) values, they gradually increased with increased doses of gamma irradiation applied to taro flour; similar results were found for blueberry by Liu et al. [29]. The observed color changes in pudding samples were likely a result of irradiation causing the degradation of anthocyanins responsible for the red color (e.g., peonidin, malvidin) and blue color (e.g., cyanidin, delphinidin) in taro flour during irradiation. Low-dose irradiation impacted the stability and antioxidant activity of plum peel anthocyanins [30]. Non-enzymatic browning reactions may further influence these color changes, according to the study by Bashir et al. [19]. These color changes may or may not impact the visual appeal and perceived quality of the pudding products, which will be discussed later.

Syneresis significantly differed among puddings with varying doses of gamma irradiation applied to taro flour; it gradually decreased with increasing irradiation dose (Table 12). This indicated that the higher irradiation doses reduced the amount of water released from the pudding during storage. The reduction in syneresis can be attributed to the gamma irradiation-induced degradation of starch molecules into simpler fragments, such as dextrins and sugars, which possess higher water affinity. This effect may also be partly related to the composition of amylopectin chains, where a higher proportion of highly branched and shorter chains confers greater water-holding capacity and slower retrogradation [31], and the reduction in apparent amylose content and the formation of lower-molecular-weight starch fragments with enhanced water-binding potential [8]. Moreover, gamma irradiation can induce the partial unfolding or denaturation of protein molecules, exposing additional hydrophilic groups and enhancing their capacity to bind water [32]. The direct characterization of irradiation-induced changes in the starch molecular structure and protein molecules should be further studied to understand how these changes affected the syneresis of pudding and similar products.

The textural properties of pudding products show a significant difference in hardness, cohesiveness, and gumminess due to varying doses of gamma irradiation (Table 13). The pudding containing gamma-irradiated taro flour at a dose of 6 kGy had the highest hardness value (1134.31 g) and gumminess value (137.56) but the lowest cohesiveness value (0.12). Gamma irradiation may have induced changes in amylose and amylopectin chains, generating smaller starch fragments with improved water-binding ability [33,34], which, in turn, support the reinforcement of the pudding’s gel network.

The use of irradiated taro flour at a dose of 6 kGy in puddings effectively reduced microbial load, resulting in significantly lower total plate counts, as well as decreased levels of yeasts and molds compared to the puddings made with non-irradiated taro flour (Table 14). Similar results were found in the study on the effects of different irradiation doses and storage periods on the microbiological characteristics of wheat (*Triticum aestivum* L.) [35].

Overall, the pudding samples were acceptable in all attributes evaluated, with the liking scores ranging from 6.70 to 8.03 (Table 15). Irradiation at the varying doses applied to taro flour did not significantly affect the appearance and color liking scores (7.53–8.03, Table 15) of the pudding samples, regardless of the observed changes in color L*a*b* values (Table 11). The texture liking scores tentatively increased (although not significantly) from 6.73 to 7.07 as the irradiation dose increased; this could have been due to the increase in texture hardness and gumminess (Table 13). Likewise, flavor and taste liking scores were overall not significantly different among samples, although there was a trend of a slight increase as the irradiation dose increased (6.97 to 7.30 and 6.83 to 7.20, respectively, Table 15). The increase in flavor liking scores was likely due to the increased pleasant, sweet volatiles (toluene, 2-cyclopenten-1-one, benzaldehyde, and 2-methyl-3-methylthio- pyrazine) and the decreased off-flavor volatiles (dimethyl disulfide, hexanal, nonanal, and octanoic acid) in the pudding sample, especially at 6 kGy (Table 9). The pudding sample containing taro flour irradiated at 6 kGy was the most liked compared to other samples (7.3 vs. 6.7–6.83); this sample received liking scores above 7.0 for all attributes evaluated. Zheng et al. [36] also reported similar positive effects of gamma irradiation on the sensory and aroma characteristics of soaked bayberry jiu.

## 4. Conclusions

The current study demonstrated the positive effects of using isomaltulose and gamma-irradiated taro flour in pudding formulations, including the delayed separation of liquid layers on the surface (syneresis) over time and the firmer texture. The taro flour that was gamma-irradiated at 6 kGy exhibited increased pleasant, sweet volatiles, which were also detected in the pudding samples. Additionally, the pudding sample containing the taro flour gamma-irradiated at 6 kGy was the most liked. This finding suggested that gamma irradiation at this specific dose of 6 kGy exerted positive effects on the sensory characteristics of the pudding. The positive influence of gamma irradiation suggested a promising avenue for improving food quality and consumer satisfaction, paving the way for a further exploration of its applications across various culinary products. Furthermore, isomaltulose, which was used in the pudding, has a lower GI compared to sucrose. This means that it causes a slower rise in blood sugar levels and can help manage blood sugar levels. It is suitable for diabetic patients and individuals who are health-conscious or aim to control their weight. Combining isomaltulose sugar with gamma-irradiated taro flour at a dose of 6 kGy appears to be a promising approach to producing a healthier pudding with improved sensory qualities and stability, making it a more appealing dessert option.

## Figures and Tables

**Table 1:** Formulations of pudding using two different types of sugar.

Ingredient (%)	Pudding Samples		
	Sucrose (S)	5% Isomaltulose (5I)	10% Isomaltulose (10I)
Lactose-free milk	65	65	62
Water	26	26	24
Sucrose	5	0	0
Isomaltulose	0	5	10
Taro flour	3	3	3
Agar	1	1	1

**Table 2:** Total soluble solids (TSS) and pH of pudding products made with different types of sugar.

Pudding Samples	TSS (°Brix)	pH
S (sugar)	16.50 ^b^ ± 0.71	6.54 ^a^ ± 0.01
5I (5% isomaltulose)	15.50 ^b^ ± 0.71	6.13 ^a^ ± 0.65
10I (10% isomaltulose)	21.50 ^a^ ± 0.71	6.51 ^a^ ± 0.06

Values are expressed as mean ± standard deviation of duplicate determinations. ^a,b^ Means within column with different superscripts are significantly different (*p* ≤ 0.05).

**Table 3:** Color characteristics of pudding products made with different types of sugar.

Pudding Samples	L* (Lightness)	a* (Redness)	b* (Yellowness)
S (sugar)	71.06 ± 1.41 ^ns^	3.34 ± 0.50 ^ns^	4.47 ± 0.73 ^ns^
5I (5% isomaltulose)	71.84 ± 0.01	3.34 ± 0.33	3.35 ± 0.23
10I (10% isomaltulose)	70.86 ± 1.12	3.08 ± 0.69	3.10 ± 1.08

Values are expressed as mean ± standard deviation of duplicate determinations. ^ns^ Means within column are not significantly different (*p* > 0.05).

**Table 4:** Syneresis (%) of pudding products made with different types of sugar after 1 and 15 days of storage.

Pudding Samples	Day 1	Day 15
S (sugar)	4.28 ± 1.30 ^ns^	11.77 ^a^ ± 0.25
5I (5% isomaltulose)	4.11 ± 0.66	11.00 ^b^ ± 0.73
10I (10% isomaltulose)	4.48 ± 0.61	10.61 ^b^ ± 0.34

Values are expressed as mean ± standard deviation of duplicate determinations. ^a,b^ Means within column with different superscripts are significantly different (*p* ≤ 0.05). ^ns^ Means within column are not significantly different (*p* > 0.05).

**Table 5:** Textural properties of pudding products made with different types of sugar.

Pudding Samples	Hardness (g)	Springiness	Cohesiveness	Gumminess (g)
S (sugar)	793.64 ^b^ ± 79.48	0.55 ± 0.07 ^ns^	0.13 ^a^ ± 0.01	106.56 ± 10.27 ^ns^
5I (5% isomaltulose)	804.10 ^b^ ± 58.47	0.54 ± 0.10	0.13 ^a^ ± 0.01	104.25 ± 10.63
10I (10% isomaltulose)	915.93 ^a^ ± 54.56	0.54 ± 0.08	0.12 ^b^ ± 0.01	109.21 ± 10.08

Values are expressed as mean ± standard deviation of duplicate determinations. ^a,b^ Means within column with different superscripts are significantly different (*p* ≤ 0.05). ^ns^ Means within column are not significantly different (*p* > 0.05).

**Table 6:** Consumer acceptance of pudding products made with different types of sugar.

Pudding Samples	Appearance	Color	Flavor	Texture	Taste	Overall Liking
S (sugar)	7.17 ^c^ ± 1.3	7.50 ± 1.4 ^ns^	6.57 ± 1.6 ^ns^	6.17 ^b^ ± 1.6	6.83 ^a^ ± 1.4	6.93 ^b^ ± 1.3
5I (5% isomaltulose)	7.23 ^b^ ± 1.3	7.47 ± 1.5	6.90 ± 1.7	5.37 ^c^ ± 1.8	4.90 ^b^ ± 2.0	5.63 ^c^ ± 1.6
10I (10% isomaltulose)	7.70 ^a^ ± 1.5	7.70 ± 1.3	6.37 ± 1.9	7.60 ^a^ ± 1.5	7.00 ^a^ ± 1.8	7.40 ^a^ ± 1.3

Values are expressed as mean ± standard deviation of duplicate determinations. ^a–c^ Means within column with different superscripts are significantly different (*p* ≤ 0.05). ^ns^ Means within column are not significantly different (*p* > 0.05).

**Table 7:** Proximate chemical composition of taro flour treated with different doses of gamma irradiation.

Dose (kGy)	Moisture (%)	Fat (%)	Protein (%)	Ash (%)	Total Carbohydrate (%)
0	5.55 ± 0.06 ^ns^	0.37 ± 0.01 ^ns^	0.74 ± 0.04 ^ns^	1.14 ± 0.06 ^ns^	92.20
2	5.40 ± 0.04	0.37 ± 0.01	0.73 ± 0.04	1.19 ± 0.01	92.31
4	5.35 ± 0.19	0.38 ± 0.01	0.73 ± 0.04	1.12 ± 0.11	92.42
6	5.36 ± 0.16	0.40 ± 0.01	0.78 ± 0.01	1.16 ± 0.07	92.30

Values are expressed as mean ± standard deviation of duplicate determinations. ^ns^ Means within column are not significantly different (*p* > 0.05).

**Table 8:** Volatile compounds of taro flours treated with different doses of gamma irradiation.

Compound	RT	Peak Area				Odor Description	References
		0 kGy	2 kGy	4 kGy	6 kGy		
Toluene	5.55	7.3 × 10^4^	6.5 × 10^4^	9.1 × 10^4^	9.8 × 10^4^	Sweet	[21]
1-Pentanol	5.64	5.3 × 10^4^	5.1 × 10^4^	5.7 × 10^4^	7.3 × 10^4^	Taro, Sweet	[22]
Furfural	6.79	6.6 × 10^4^	5.7 × 10^4^	ND	ND	Bread, Almond, Sweet	[23]
2-Cyclopenten-1-one	6.80	1.2 × 10^4^	1.1 × 10^5^	2.6 × 10^5^	2.9 × 10^5^	Jasmine, Sweet	[24]
Butyrolactone	8.00	9.7 × 10^4^	1.9 × 10^5^	1.6 × 10^5^	1.4 × 10^5^	Caramel, Sweet	[23]
Benzaldehyde	8.64	3.7 × 10^5^	5.4 × 10^5^	7.5 × 10^5^	1.4 × 10^6^	Almond, Nutty	[23]
Hexanoic acid	8.87	7.7 × 10^5^	5.6 × 10^5^	4.9 × 10^5^	2.9 × 10^5^	Sweat, Cheese	[23]
Vanillin	12.99	2.2 × 10^6^	1.9 × 10^6^	9.1 × 10^5^	1.9 × 10^6^	Vanilla, Sweet	[23]

Note: ND means not detected.

**Table 10:** Total soluble solids (TSS) and pH of pudding products containing taro flour treated with different doses of gamma irradiation.

Dose (kGy)	TSS (°Brix)	pH
0	21.67 ± 0.52 ^ns^	6.50 ± 0.03 ^ns^
2	22.00 ± 0.00	6.50 ± 0.02
4	21.67 ± 0.52	6.52 ± 0.03
6	22.00 ± 0.63	6.48 ± 0.03

Values are expressed as mean ± standard deviation of duplicate determinations. ^ns^ Means within column are not significantly different (*p* > 0.05).

**Table 11:** Color characteristics of pudding products containing taro flour treated with different doses of gamma irradiation.

Dose (kGy)	L*	a*	b*
0	71.88 ± 1.36 ^ns^	3.78 ^a^ ± 0.17	3.78 ^b^ ± 0.22
2	71.71 ± 2.13	3.26 ^b^ ± 0.26	4.14 ^a^ ± 0.13
4	71.84 ± 0.86	3.28 ^b^ ± 0.39	4.41 ^a^ ± 0.31
6	71.52 ± 0.94	3.86 ^a^ ± 0.53	4.33 ^a^ ± 0.20

Values are expressed as mean ± standard deviation of duplicate determinations. ^a,b^ Means within column with different superscripts are significantly different (*p* ≤ 0.05). ^ns^ Means within column are not significantly different (*p* > 0.05).

**Table 12:** Syneresis of pudding products containing taro flour treated with different doses of gamma irradiation.

Dose (kGy)	Day 1	Day 15
0	5.58 ^a^ ± 0.68	11.64 ^a^ ± 0.22
2	5.13 ^b^ ± 0.72	9.45 ^b^ ± 0.41
4	5.03 ^b^ ± 0.59	9.39 ^b^ ± 0.31
6	4.89 ^b^ ± 0.25	9.03 ^b^ ± 0.41

Values are expressed as mean ± standard deviation of duplicate determinations. ^a,b^ Means within column with different superscripts are significantly different (*p* ≤ 0.05).

**Table 13:** Textural properties of pudding products containing taro flour treated with different doses of gamma irradiation.

Dose (kGy)	Hardness (g)	Springiness	Cohesiveness	Gumminess (g)
0	890.43 ^c^ ± 141.01	0.51 ± 0.07 ^ns^	0.14 ^a^ ± 0.02	121.82 ^b^ ± 12.12
2	1004.56 ^b^ ± 111.20	0.53 ± 0.08	0.13 ^ab^ ± 0.02	126.82 ^b^ ± 12.15
4	1025.89 ^b^ ± 94.54	0.52 ± 0.06	0.13 ^ab^ ± 0.02	128.63 ^b^ ± 7.22
6	1134.31 ^a^ ± 48.53	0.52 ± 0.06	0.12 ^b^ ± 0.01	137.56 ^a^ ± 13.85

Values are expressed as mean ± standard deviation of duplicate determinations. ^a–c^ Means within column with different superscripts are significantly different (*p* ≤ 0.05). ^ns^ Means within column are not significantly different (*p* > 0.05).

**Table 14:** Microbial analysis of pudding products containing taro flours gamma-irradiated at 0 and 6 kGy.

Dose (kGy)	Total Plate Count (CFU/g)		Yeasts and Molds (CFU/g)	
	Day 0	Day 7	Day 0	Day 7
0	45 ^a^ ± 3	120 ^a^ ±10	<10 ^ns^	<10 ^ns^
6	30 ^b^ ± 4	<10 ^b^	<10	<10

Values are expressed as mean ± standard deviation of duplicate determinations. ^a,b^ Means within column with different superscripts are significantly different (*p* ≤ 0.05). ^ns^ Means within column are not significantly different (*p* > 0.05).

**Table 15:** Consumer acceptance of pudding products containing taro flour treated with different doses of gamma irradiation.

Dose (kGy)	Appearance	Color	Flavor	Texture	Taste	Overall Liking
0	7.73 ± 1.2 ^ns^	7.60 ± 1.0 ^ns^	6.97 ^ab^ ± 1.5	6.73 ± 1.4 ^ns^	6.83 ± 1.7 ^ns^	6.90 ^b^ ± 1.3
2	7.83 ± 1.2	7.53 ± 1.1	6.80 ^b^ ± 1.1	6.83 ± 1.5	6.77 ± 1.4	6.70 ^b^ ± 1.1
4	8.03 ± 1.0	7.83 ± 1.02	6.87 ^ab^ ± 1.2	6.87 ± 1.4	6.70 ± 1.3	6.83 ^b^ ± 1.3
6	7.97 ± 1.0	7.90 ± 1.0	7.30 ^a^ ± 1.0	7.07 ± 1.2	7.20 ± 1.2	7.30 ^a^ ± 1.2

Values are expressed as mean ± standard deviation of duplicate determinations. ^a,b^ Means within column with different superscripts are significantly different (*p* ≤ 0.05). ^ns^ Means within column are not significantly different (*p* > 0.05).

## Data Availability

The original contributions presented in the study are included in the article; further inquiries can be directed to the corresponding author.
